# Evidential requirements for the regulatory hazard and risk assessment of respiratory sensitisers: methyl methacrylate as an example

**DOI:** 10.1007/s00204-023-03448-w

**Published:** 2023-02-17

**Authors:** Bette Meek, James W. Bridges, Andrew Fasey, Ursula G. Sauer

**Affiliations:** 1 University of Ottawa, Ottawa, Canada; 2 Emeritus Professor, University of Surrey, Guildford, Surrey UK; 3 PTK (Finland) Ltd., Helsinki, Finland; 4 Scientific Consultancy–Animal Welfare, Hallstattfeld 16, 85579 Neubiberg, Germany

**Keywords:** Respiratory sensitisation, Harmonised classification and labelling (CLH), Weight-of-evidence (WoE), Modified Bradford Hill considerations, Mode-of-action (MoA)

## Abstract

This review addresses the need for a framework to increase the consistency, objectivity and transparency in the regulatory assessment of respiratory sensitisers and associated uncertainties. Principal issues are considered and illustrated through a case study (with methyl methacrylate). In the absence of test methods validated for regulatory use, formal documentation of the weight-of-evidence for hazard classification both at the level of integration of individual studies within lines of evidence and across a broad range of data streams was agreed to be critical for such a framework. An integrated approach is proposed to include not only occupational studies and clinical evidence for the regulatory assessment of respiratory sensitisers, but also information on structure and physical and chemical factors, predictive approaches such as structure activity analysis and in vitro and in vivo mechanistic and toxicokinetic findings. A weight-of-evidence protocol, incorporating integration of these sources of data based on predefined considerations, would contribute to transparency and consistency in the outcome of the assessment. In those cases where a decision may need to be taken on the basis of occupational findings alone, conclusions should be based on transparent weighting of relevant data on the observed prevalence of occupational asthma in various studies taking into account all relevant information including the range and nature of workplace exposures to the substance of interest, co-exposure to other chemicals and study quality.

## Introduction

From a scientific perspective, respiratory sensitisation (clinically manifested often as “asthma”) is defined as a state of heightened sensitivity and/or responsiveness of the respiratory tract to a specific allergen resulting from prior exposure (elicitation) and immunological priming (sensitisation) (Cochrane et al. 2015). By comparison, the regulatory definitions for respiratory sensitisation provided in the European Union (EU) Classification, Labelling and Packaging (CLP) Regulation (European Parliament (EP) and Council 2008) or in the European Chemicals Agency (ECHA) Endpoint-Specific Guidance on Information Requirements and Chemical Safety Assessment (ECHA 2017a) do not distinguish between immunological and non-immunological mechanisms of respiratory sensitisation. In the ECHA (2017a) Guidance, it is stated: “A respiratory sensitiser is an agent that will lead to hypersensitivity of the airways following inhalation exposure to that agent. Respiratory sensitisation (or hypersensitivity) is a term that is used to describe asthma and other related respiratory conditions (rhinitis, extrinsic allergic alveolitis), irrespective of the mechanism (immunological or non-immunological) by which they are caused. In contrast, skin allergy is based on an immunological mechanism.”

A broad spectrum of in chemico, in vitro and in vivo test methods to assess skin sensitisation and irritation has been validated and adopted for regulatory use (Gilmour et al. 2020). However, there are no test methods to assess the potential for respiratory sensitisation that have been validated for regulatory use despite years of research into this area (Arts 2020). The determination that a substance is a respiratory sensitiser has, in practice, generally been based on reliable occupational health evidence and clinical experience (e.g. Arts 2020).

The interpretation of results of human case reports and epidemiological studies for assessment of individual chemicals is often complicated by co-exposures to complex mixtures of toxic and non-toxic substances, for which composition varies (Goldberg 2007). Also, epidemiological studies relevant to consideration of respiratory sensitisers often do not include reliable exposure estimates or descriptions of the specific substances or mixtures to which the workers were exposed, nor do they always fully address potentially confounding factors (Swaen 2006). These considerations, together with the lack of accepted test methods to assess (or exclude) the potential to cause immunological sensitisation leading to respiratory reactions, complicate the assessment of respiratory sensitisers.

This review addresses relevant considerations to make recommendations to enhance the transparency, consistency and objectivity of the assessment of respiratory sensitisers and associated uncertainties.

The content builds upon discussions held in October 2021 of an independent Expert Panel review commissioned by the European Chemical Industry Council (Cefic) Methacrylates Sector Group and the Methacrylate Producers Association, Inc. The independent Expert Panel review addressed questions related principally to the evidence base for methyl methacrylate (MMA). After the Panel meeting, the experts considered these questions in the context of broader aspects relevant to the identification and characterisation of respiratory sensitisers. The observations and recommendations emanating from these broader discussions are presented in “Issues to be considered for respiratory sensitisation assessment”, referencing evidence available for MMA in “Classification of respiratory sensitisers” as an example of the nature of datasets commonly encountered in assessing the potential of substances to cause respiratory sensitisation (see Appendix before the Bibliography for Glossary with definition of key terms). Finally, “Recommendations from the Expert Panel: Regulatory hazard and risk assessment of respiratory sensitisers” summarises recommendations deriving from the outcome of the review.

## Issues to be considered for respiratory sensitisation assessment

### Pathophysiology of occupational asthma and of allergic reactions

As a starting point for the further review, this section considers scientific evidence on the pathophysiology of occupational asthma (OA), as an important clinical manifestation of respiratory sensitisation in the occupational setting, and on the pathophysiology of allergic reactions. OA is defined as inflammation of the airways that is causally related to exposure in the working environment to specific airborne dusts, gases, vapours or fumes (Cartier 1994; Mims 2015).

Focus of the review is on respiratory effects caused by low molecular weight substances (< 900 Da) since they often have the potential to cause allergic contact dermatitis (Krutz et al. 2021). Also, the effect pattern of low molecular weight respiratory sensitisers is distinct from that of high molecular weight respiratory sensitisers (Vandenplas et al. 2019) though the underlying mechanisms are not yet fully understood.

Generally, OA can be induced by an immune reaction but also by irritation, neurosensory events or pharmacological events (De Vooght et al. 2010; Tarlo and Lemiere 2014). To date, it is not fully understood how low molecular weight substances react in the lung to initiate OA. These knowledge gaps result in uncertainty in the distinction of allergic asthma from non-allergic asthma. For this reason, the definitions for respiratory sensitisation provided in the EU CLP Regulation (EP and Council 2008) and in the ECHA (2017a) Endpoint-Specific Guidance purposely do not distinguish between OA induced by sensitisation vs. irritation, as introduced above.

By comparison, it is known that OA caused by allergic reactions to high molecular weight substances is mediated by type 1 allergy with specific immunoglobulin E (IgE) leading to classical T-helper cell 2 inflammatory pathways. However, the importance of this mechanism for OA caused by low molecular weight substances is in doubt because IgE sensitisation against the offending substances can often not be demonstrated, either in patients or in experimental models. Rather, available data indicate that the traditional paradigm of type 1 allergy does not universally apply to substance-induced OA and less well-characterised immune pathways presumably also play a role (De Vooght et al. 2010; Baur et al. 2019).

These fundamental knowledge gaps complicate, then, the assessment of whether a substance causes the development of OA as opposed to the aggravation of pre-existing or coincidentally acquired asthma.

Experimental and clinical evidence indicates that sensitisation can occur in parts of the body distinct from the site of elicitation (Tsui et al. 2020). On this basis, any substance that can induce sensitisation ought to be considered a potential ‘sensitiser’ in qualitative hazard assessment, regardless of the target organ (i.e. where clinical manifestations of sensitisation occur upon elicitation) (Thá et al. 2021). Sensitisers that can be inhaled can in principle, then, also cause allergic asthma (or respiratory manifestations) in a previously sensitised individual. However, this contrasts with evidence that only some of the many substances that cause dermal contact allergies also cause OA. As per Kimber et al. (2014), less than eighty of the thousand or more known dermal sensitisers (8%) have been confirmed as respiratory sensitisers. This low proportion of confirmed respiratory sensitisers may be due to limited potential for inhalation exposure to many dermal sensitisers or to differential sensitivities of target organs. In contrast, it has been proposed that respiratory sensitisers are most often also potent skin sensitisers (see e.g. Dearman et al. 2013).

The low proportion of confirmed respiratory sensitisers as compared with dermal sensitisers may also be a function of variations in the nature or number of receptor sites to which the respective substance may bind in various organs. For example, Lalko et al. (2012) suggested that the distinction between dermal and respiratory sensitisers might be established by the type of protein interaction that leads to the immune reaction. This can be evaluated e.g. in the Organisation for Economic Co-operation and Development (OECD) Test Guideline (TG) 442C Direct Peptide-Reactivity Assay, which is an in chemico assay. Specifically, Lalko and co-workers suggested that if a substance preferentially reacts with cysteine in the Direct Peptide-Reactivity Assay, it will more likely cause skin sensitisation, and if it preferentially reacts with lysine, it will more likely cause respiratory sensitisation (Lalko et al. 2012). Notably, however, in a critical evaluation of Direct Peptide-Reactivity Assay data for 200 chemicals (15 respiratory sensitisers, 129 skin sensitisers, 57 non-sensitisers), Krutz et al. (2021) reported that the reactivity of the low molecular weight respiratory sensitisers for lysine residues was threefold higher than for skin sensitisers, but that this difference was driven largely by the high representation of acid anhydrides among the respiratory sensitisers with clear selectivity for lysine (Krutz et al. 2021). Sadekar et al. (2021) cautioned that any such results might be biassed due to the very limited dataset for low molecular weight respiratory sensitisers. Also, Arts (2020) noted that measurements of the preferential reactivity of a substance with either lysine or cysteine does not provide conclusive evidence for either the presence or absence of respiratory sensitisation potential.

The Panel agreed that with currently available testing and assessment methodology, it is difficult to distinguish between irritation, sensitisation and neurosensory reactions in the respiratory tract and noted also that these mechanisms may not be necessarily mutually exclusive.

Further, the Panel was unaware of any robust toxicological testing approach to clearly distinguish between irritant and immune-related pulmonary reactions, although well-designed experiments combining immunological and physiological readouts in animal models could shed light on these issues (Vanoirbeek et al. 2003, 2006). An approach for a prospective animal assay has been proposed (Pollaris et al. 2019). In this test, animals that were sensitised via the dermal route are challenged via intranasal instillation. Readouts include immune-related parameters in bronchoalveolar lavage and lung tissue, blood, cervical and auricular lymph nodes, as well as pulmonary physiological responses, including bronchial hyperreactivity to methacholine. Lack of such responses in sensitised animals may be interpreted as evidence that the substance has low potential to induce respiratory sensitisation.

### Human data for the assessment of respiratory sensitisers

In the absence of in vivo (or in vitro) test methods to assess the potential for respiratory sensitisation validated for regulatory use (Arts 2020), the assessment of whether a substance has the potential to cause respiratory sensitisation will generally rely on human data. The need for reliance on human data is acknowledged with provisions from the ECHA (2017a) Endpoint-Specific Guidance indicating: “Although predictive models are under validation, there is as yet no internationally recognised animal method for identification of respiratory sensitisation. Thus, human data are usually evidence for hazard identification. In case existing human data are available on respiratory sensitisation, those data should be assessed and included in the IUCLID [International Uniform Chemical Information Database] *dossier*” (ECHA 2017a; Chapter R.7.3.10.2). Similarly, the ECHA (2017b) Guidance on Application of CLP Criteria states: “Substances shall be classified as respiratory sensitisers if there is evidence in humans or other sufficient evidence, including read-across that the substance can lead to specific respiratory hypersensitivity” (ECHA 2017b; Chapter 3.4.2.1.3.1).

Human data for the assessment of respiratory sensitisers can be derived from e.g. epidemiological studies such as worker health studies, case reports submitted to national health surveillance databases and/or specific inhalation challenge (SIC) testing. The ECHA (2017a) Endpoint-Specific Guidance also acknowledges limitations of human respiratory sensitisation data: “Although human studies may provide some information on respiratory hypersensitivity, the data are frequently limited and subject to the same constraints as human skin sensitisation data” (ECHA 2017a; Chapter R.7.3.10.2).

SIC tests include the controlled exposure of a patient, under laboratory conditions, to an agent encountered in his or her workplace. This includes a control challenge, a gradual increase of exposure to the suspected agent and close monitoring of the patient during the challenge and for at least six hours afterwards. A positive response is defined by a fall in forced expiratory volume in 1 s ≥ 15% from baseline (Vandenplas et al. 2014). SIC tests are generally considered the reference standard to diagnose the causative agent of OA in individual patients (Vandenplas et al. 2014). The controlled experimental protocols are performed only in a few specialised hospitals. In both North America and Europe, working groups have prepared guidance for the conduct of SIC tests, including diagnosis of OA (see e.g. Tarlo et al. 2008; Vandenplas et al. 2014). Concentrations applied in the SIC tests should reflect real-life exposure conditions. Often, however, it is not known to which specific substances workers were exposed since substance characterisation may be difficult to undertake in the clinical setting. Nonetheless, the Consensus Statement of the American College of Chest Physicians on Diagnosis and Management of Work-Related Asthma states that “a challenge chamber or closed-circuit apparatus can be used to generate and monitor the suspected agent” (page S24 in Tarlo et al. 2008).

The objective of SIC testing is to determine whether pulmonary function has been impaired by exposure to the test agent rather than to distinguish mechanisms of effect, since many sensitisers are also irritants (e.g. di-isocyanates). The Panel agreed that SIC tests do not enable decisions to be made on whether a positive response is more likely due to an immune reaction or to irritation (further discussed in  “Mechanisms of respiratory sensitisation”).

### Prevalence / incidence of occupational asthma in epidemiological studies

The ECHA (2017a) Endpoint-Specific Guidance indicates which key elements of human studies need to be recorded when such data are used for regulatory purposes: “*For evaluation purposes, existing human experience data for respiratory sensitisation should contain sufficient information about:*

- The test protocol used (study design, controls)
- The substance or preparation studied (should be the main, and ideally, the only substance or preparation present which may possess the hazard under investigation)
- The extent of exposure (magnitude, frequency and duration)
- The frequency of effects (versus number of persons exposed)
- The persistence or absence of health effects (objective description and evaluation)
- The presence of confounding factors (e.g. pre-existing respiratory health effects, medication; presence of other respiratory sensitisers)
- The relevance with respect to the group size, statistics, documentation
- The healthy worker effect” (ECHA 2017a; Chapter R.7.3.10.2)

The Panel noted that reliable information on the frequency (incidence) of reported effects in exposed populations was an important element for the assessment of respiratory sensitisers.

A comprehensive evaluation of the incidence of OA caused by a particular substance should also consider the impact of the “healthy worker” effect (Baillargeon 2001) on reported low incidences. For example, workers with pre-existing or de novo asthma might resign due to the strong odour of a substance before or after developing OA. Further, workers may develop asthma unrelated to their workplace (see e.g. Nemery 2004).

### Mechanisms of respiratory sensitisation

Regarding attempts to differentiate between irritant and immune-related pulmonary reactions in humans, the only available diagnostic tool is the SIC test, which as noted above also has limitations (“Human data for the assessment of respiratory sensitisers”). Performing bronchoalveolar lavages after the SIC test was considered by the Panel as potentially helpful in distinguishing between immune and irritant reactions (by informing on eosinophilia or an increase in specific cytokines in the lung). However, bronchoalveolar lavage requires bronchoscopy, and such invasive procedure is not justified for assessing responses to SIC testing. Assessing inflammatory responses in induced sputum has also been used during SIC testing (Lemiere 2007).

Regarding neurosensory reactions, asthmatics do not necessarily exhibit bronchoconstriction when they are confronted with strong odours or irritants. Nonetheless, bronchospasms sometimes develop in anticipation of the asthmatic reaction. Nociceptors can trigger asthmatic reactions, and there is evidence that they react to strong fragrances and spices (Jaén and Dalton 2014; Vlemincx et al. 2021). It follows that there could be neurosensory mechanisms by which irritants and strong fragrances might trigger asthmatic responses without involvement of an immune reaction. However, there is also increasing evidence of neuro-immune crosstalk being involved in allergic inflammation (Kabata and Artis 2019).

The Panel noted that symptoms observed in humans are not necessarily informative with respect to mechanism of induction. Patients that are exposed to airborne sensitisers may develop conjunctivitis and rhinitis. These symptoms may also occur in patients exposed to irritant substances by inhalation. It was discussed that sensitisation takes time (weeks to years) to evolve. Generally, immune sensitisation is considered more likely if the patient has an asymptomatic latency period preceding development of symptoms or if low exposures that do not affect fellow workers cause symptoms (Legiest and Nemery 2012). If, however, a patient shows symptoms after first contact, this is most likely an irritant reaction, unless there is cross-sensitisation with an agent to which the individual is already sensitised.

The Panel also noted that when asthmatic responses are observed upon exposure to high levels of a respiratory irritant, it cannot be excluded with confidence that the asthmatic response indicates causation and not merely provocation. It would be unethical to provoke a reaction in asthmatic patients who did not have prior exposure to a known respiratory sensitiser (except in well-designed studies and with approval of an ethics committee).

### Formal weight-of-evidence (WoE) evaluation to support the classification of respiratory sensitisers

#### Background to WoE evaluations

There is no single test method (or tiered testing strategy) that can provide conclusive evidence to support the classification and labelling of a substance as a respiratory sensitiser (“Regulatory provisions implemented in the EU for the classification of respiratory sensitisers”). Therefore, the corresponding criteria provided in the EU CLP Regulation (EP and Council 2008) emphasise the need to consider all available evidence in a weight-of-evidence (WoE) evaluation (Box 1). The WoE has been defined as “*the extent to which evidence supports possible answers to a scientific question*”, and the WoE assessment is “*a process in which evidence is integrated to determine the relative support for possible answers to a scientific question*” (EFSA SC 2017). In WoE evaluations, lines of evidence conjoin different studies and/or different types of studies (as relevant for the formulated hypothesis) to critically evaluate an underlying hypothesis (Bridges et al. 2017).

**Figure:**
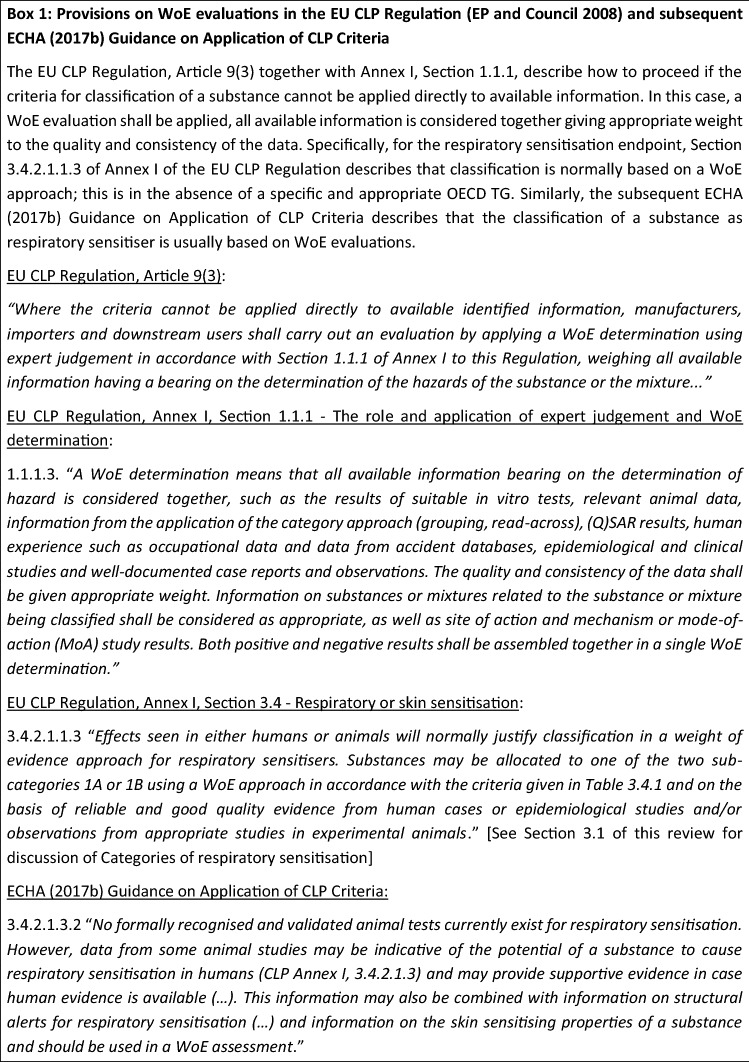

Formal WoE evaluation frameworks have been developed with the objective to increase transparency, consistency and objectivity on how the available data were identified, selected and organised, how they were assessed within lines of evidence and how they were integrated across these different lines of evidence as a basis for conclusion. While applied principally in the consideration of data on effects related to chronic, repeated exposure, the application of formal, objective and transparent WoE considerations to respiratory sensitisation has potential to increase the consistency and defensibility of expert judgements concerning the comparative extent of the evidence to support regulatory classification and assessment for this endpoint.

The internationally agreed World Health Organisation—International Programme on Chemical Safety (WHO IPCS) Framework on Mode-of-Action (MoA) and Species Concordance Analysis (Sonich-Mullin et al. 2001; Boobis et al. 2006; Meek et al. 2014a, b) describes how WoE evaluations can be conducted using considerations modified from those first introduced by Sir Bradford Hill to assess causality in epidemiological studies (Hill 1965). The modified Bradford Hill considerations presented in the WHO IPCS Framework on MoA and Species Concordance Analysis include (1) biological concordance; (2) essentiality of key events; (3) concordance of empirical observations among key events (related to dose–response, temporality of key events and the incidence of key events as compared with the adverse effect); (4) consistency among different biological contexts; and (5) analogy across chemicals (Box 2, adapted from Meek et al. 2014b). Hence, the modified Bradford Hill considerations address the concordance and consistency of the evidence across different lines of evidence and levels of biological organisation for mechanistic hypotheses. A relevant subset of these considerations has also been adopted by the OECD for the assessment of chemical agnostic adverse outcome pathways (AOPs; OECD AOP Developers’ Handbook; OECD 2022).

**Figure:**
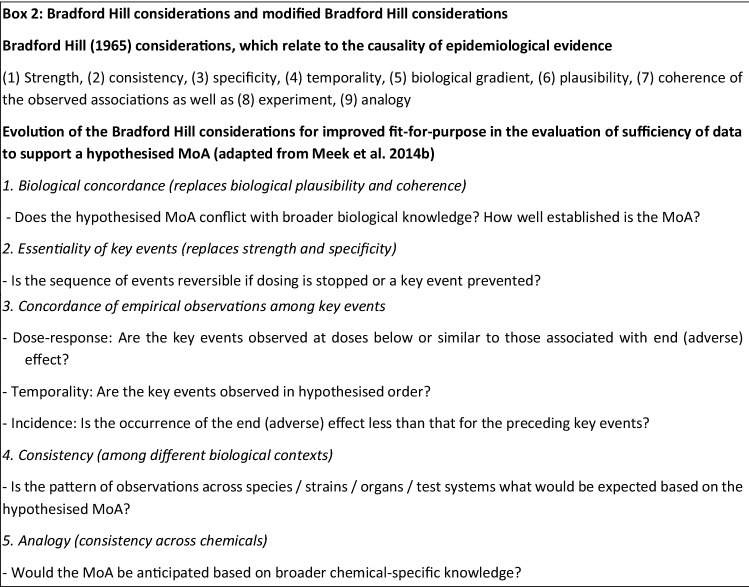

The WHO IPCS Framework on MoA and Species Concordance Analysis is also considered in the templates for WoE and uncertainty evaluation in risk assessment prepared by ECHA (https://www.echa.europa.eu/web/guest/support/guidance-on-reach-and-clp-implementation/formats; accessed November 2022). These ECHA WoE / uncertainty templates, that further take into account guidance from OECD on AOPs and integrated approaches to testing and assessment (OECD 2016a, b, 2017a, b, 2020), include six sections on:

1. Problem formulation
2. Search strategy and documentation of all information
3. Assessment of quality of individual evidence
4. Integration and weighing of evidence making use of the modified Bradford Hill considerations
5. Uncertainty analysis
6. Conclusions

While degrees of prescription in WoE frameworks vary, formal analysis frames the basis for consistent and transparent consideration of important aspects of evaluation of the appropriate contribution of studies, lines of evidence and the overall database to conclusions. Therefore, they can be adapted to the needs of the given evaluation. For example, the anticipated pattern of dose–response and temporal concordance for respiratory sensitisation needs to be taken into account. Application of a formal WoE evaluation helps the reader to clearly understand the approach taken as well as the underlying rationale. It also requires delineation of uncertainties based on limitations of the data and provides transparency on how these uncertainties are being considered in drawing a conclusion and the associated level of confidence.

#### Application of formal WoE frameworks for the assessment of respiratory sensitisers

The Panel agreed that formal WoE evaluation would contribute to increasing transparency, consistency and objectivity in drawing conclusions on the classification of respiratory sensitisers and in identifying and addressing underlying uncertainties, taking into account different types of human data, results from in vitro and in vivo toxicity tests, in silico models such as quantitative structure–activity relationships (QSARs) and inference from similar substances (read-across). Structural, physico-chemical information and toxicokinetic data for the relevant and related substances should also be considered.

Formal WoE evaluations for respiratory sensitisation also require explicit consideration of the relevance, reliability and weighting of contributing studies. This is reflected in *Assessment of quality of individual evidence* of the ECHA WoE / uncertainty templates (see “Background to WoE evaluations” above). Evaluation considerations for case reports and clinical studies need to clarify the extent to which each report and study contributes to the overall evaluation within a line of evidence. Similarly, it needs to be defined, in advance, how the different types of information should be integrated across lines of evidence. This is reflected in  Sect. *WoE analysis—application of levels of confidence* of the ECHA WoE/uncertainty templates. Predefinition of relevant WoE considerations should also consider the unique nature of the endpoint respiratory sensitisation, including, for example, variation in individual susceptibility relevant to the concordance of dose–response and temporal relationships.

Use of predefined, contextually relevant considerations contributes to increasing transparency, consistency and objectivity in selecting studies and lines of evidence, in establishing the relevance and reliability of the data within a line of evidence (e.g. human, animal, in vitro) and in weighting and integrating the data from all lines of evidence. Integration across different lines of evidence takes into consideration human data from e.g. worker health (i.e. epidemiological) studies, case reports, information from national health surveillance databases, data from animal studies and in vitro assays as well as in silico and computational prediction tools to more robustly inform outcome, etc. In evaluating the evidence from different (types of) human studies and data sources, the prevalence and incidence from different populations and sectors should be compared and weighted while also considering whether study methodology and estimation of prevalence values have been adequately reported. The weighting and integration of data from all lines of evidence can also support the justification to focus the WoE evaluation on a limited number of studies that are considered most relevant and reliable or to conduct the WoE evaluation more broadly across a range of acceptable studies.

The modified Bradford Hill consideration ‘biological concordance’ addresses questions related to the substance’s MoA (Box 2). Regarding the scientific evidence available on mechanisms that may be involved in the development of OA, the Panel noted that it is difficult to distinguish between irritation, sensitisation and neurosensory reactions in the respiratory tract (“Mechanisms of respiratory sensitisation”). However, the classification of respiratory sensitisers currently does not require evidence of an immunological mechanism of hypersensitivity (“Regulatory provisions implemented in the EU for the classification of respiratory sensitisers”).

Consideration of the concordance of dose–response relationships across different levels of biological organisation and data sources is an important component of integration of lines of evidence in formal WoE evaluation frameworks (see, for example, the European Food Safety Authority (EFSA) and ECHA (2018) Guidance for the identification of endocrine disruptors in the context of Regulations (EU) No 528/2012 and (EC) No 1107/2009). The modified Bradford Hill consideration ‘concordance of dose–response relationships’ addresses the extent to which the pattern of dose–response relationships for key events is consistent with what would be anticipated, based on the hypothesised MoA or AOP. Thereby, considerations on the concordance of dose–response relationships across different data sources and lines of evidence also support the evaluation whether the effects observed in (selected) case reports are causally linked to exposure to the substance of interest. Considerations on dose–response relationships for sensitisation need to consider evidence that the immune reaction that leads to sensitisation may decrease at higher and/or more prolonged or repeated exposures (Vanoirbeek et al. 2009). It is not yet understood why this occurs; continuous exposure may lead to tolerance.

The concordance of temporal relationships also needs to be considered in the context of what is expected for elicitation and sensitisation. Since sensitisation takes time (weeks to years) to evolve (“Mechanisms of respiratory sensitisation”), information on the onset of symptoms after first contact could be useful to establish the temporality of events. Similarly, a comparison of the confidence in the evidence available for the substance of interest with that for known respiratory sensitisers will be informative for formal WoE evaluations (addressed in the modified Bradford Hill consideration ‘analogy’).

Finally, while expert judgement cannot be fully codified, the application of formal evaluation frameworks provides a structured approach to evaluate whether the WoE supports a hypothesis that the substance of interest causes the development of OA. This enforces discipline and improves consistency in conducting the evaluation through delineation of predefined considerations, drawing upon prior collective experience.

## Classification of respiratory sensitisers

### Regulatory provisions implemented in the EU for the classification of respiratory sensitisers

Table 3.4.1 of Annex I of the EU CLP Regulation (EP and Council 2008) presents the Hazard Categories for respiratory sensitisers:

“*Substances shall be classified as respiratory sensitisers (Category 1) where data are not sufficient for sub-categorisation in accordance with the following criteria: (a) if there is evidence in humans that the substance can lead to specific respiratory hypersensitivity; and/or (b) if there are positive results from an appropriate animal test.*

- *Subcategory 1A: Substances showing a high frequency of occurrence in humans; or a probability of occurrence of a high sensitisation rate in humans based on animal or other tests (Footnote 1). Severity of reaction may also be considered.*
- *Subcategory 1B: Substances showing a low to moderate frequency of occurrence in humans; or a probability of occurrence of a low to moderate sensitisation rate in humans based on animal or other tests*^*1*^*. Severity of reaction may also be considered*.”

Footnote 1 to Table 3.4.1 of Annex I of the EU CLP Regulation summarises the *status quo* regarding test method availability: “*At present, recognised and validated animal models for the testing of respiratory hypersensitivity are not available. Under certain circumstances, data from animal studies may provide valuable information in a WoE assessment*.”

Neither the EU CLP Regulation nor the subsequent ECHA (2017a, b) guidance include specific test methods or specific data thresholds to establish that a substance is a respiratory sensitiser. Therefore, expert judgement based on a WoE approach is crucial to determine whether the available evidence supports the conclusion that a substance should be classified as a respiratory sensitiser.

The ECHA (2017a) Endpoint-Specific Guidance, Chapter R.7.3.10.2 on respiratory sensitisation, states that the evaluation of human data requires *sufficient* information on the test protocol used, on the identity of the substance, the extent of exposure (magnitude, frequency, duration), frequency of effects, persistence or absence of health effects, confounding factors, relevance of the data with respect to group size, statistics and documentation as well as the healthy worker effect (“Prevalence / incidence of occupational asthma in epidemiological studies”). The ECHA (2017a) guidance also indicates that evidence that significant occupational exposure has *not* resulted in respiratory allergy may serve as a basis to conclude that a substance lacks the potential for sensitisation: “…*where there is reliable (e.g. supported by medical surveillance reports) evidence that a large cohort of subjects has had opportunity for regular significant inhalation exposure to a substance for a sustained period of time in the absence of respiratory symptoms, or related health complaints, then this will provide reassurance within a WoE approach regarding the absence of a respiratory sensitisation hazard*” (ECHA 2017a).

Further, as per Sect. 3.4.2.1.2.1 of Annex I of the EU CLP Regulation, the presence of immunological mechanisms does not have to be demonstrated to support the classification as respiratory sensitiser. Thereby, the CLP criteria for respiratory sensitisation are not clearly demarcated from the criteria to classify a substance for respiratory tract irritation (Sect. 3.8.2.2.1 in Annex I of the EU CLP Regulation), i.e. “*respiratory irritant effects (characterised by localised redness, oedema, pruritis and/or pain) that impair function with symptoms such as cough, pain, choking, and breathing difficulties are included*”. Similarly, the ECHA (2017a) Endpoint-Specific Guidance states that respiratory sensitisation also includes asthma that is caused by non-immunological mechanisms (see above  “ Introduction”).

### Example Case Study: methyl methacrylate (MMA)

#### Background on use and hazard profile

MMA (C_5_H_8_O_2_; Chemical Abstracts Service (CAS) No. 80–62-6) is an α,β-unsaturated ester monomer that is used widely to produce polymers for a wide range of industrial and consumer applications including acrylic sheets, injection moulding and extrusion, paints and coatings, adhesives and sealants, nonwovens, paper, print and packaging, construction and textiles. Some MMA is supplied in the form of two-part (monomer and polymer) dental and surgical cements (Borak et al. 2011; US EPA 2016). Borak et al. (2011) estimated that more than 15,000 industrial workers worldwide were employed in facilities producing MMA and MMA-based polymers. Comprehensive data on the numbers of workers employed in downstream industries that handle MMA are not available, although they are expected to be considerably higher. For example, the dental sector alone comprises approx. 210,000 dental technicians and 280,000 dentists within the EU (FEPPD 2021). It is important to note that consumers are generally not exposed to MMA.

MMA causes both irritation of the skin and respiratory tract in laboratory animals and exposed humans (Borak et al. 2011). At high concentrations (approx. 200 ppm and higher, depending on the study), MMA causes damage to the rodent upper respiratory tract, in both acute and repeated-dose toxicity tests (reviewed by Pemberton et al. 2013). Also, MMA has been reported to cause discomfort and irritation in workers in the cast acrylic sheets industry (see e.g. Pickering et al. 1993).

On account of its respiratory irritation potential, and further due to its strong acrid, fruity odour, MMA also has the potential to cause neurosensory effects. Reported odour thresholds for MMA are 0.049 ppm for detection and 0.34 ppm for recognition (US EPA 2008; referring to data from the American Industrial Hygiene Association). In humans, local neurological effects have been reported upon exposure to products containing MMA (Leggat and Kedjarune 2003; Leggat et al. 2009). Finally, acrylates, and MMA specifically, have been associated with OA in the clinical literature (Walters et al. 2017; Suojalehto et al. 2020). These findings have raised the question whether MMA might cause respiratory sensitisation.

The Panel considered whether MMA has the potential to induce an immune reaction – in any target organ. As regards the potential of MMA to induce immune reactions in the skin, the Panel concluded that there is an abundance of in vitro, in vivo and human data available addressing almost all key events in the OECD AOP for skin sensitisation (https://aopwiki.org/wiki/index.php/Aop:40). Thus, available data indicate that MMA is a dermal sensitiser, albeit a weak one. For example, in the Local Lymph Node Assay (OECD TG 429), the EC3 value for MMA (dissolved in pure acetone) was 90% (Betts et al. 2006); see Glossary in the Appendix for definition and interpretation of the EC3 value. Further, MMA consistently reacts preferentially with cysteine (Wareing et al. 2017; MPA 2020; OECD 2021), which may indicate that MMA is more likely to cause skin sensitisation than respiratory sensitisation (“ Pathophysiology of occupational asthma and of allergic reactions”).

The overall conclusion that MMA is a weak dermal sensitiser can likely be explained by two physico-chemical properties i.e. (1) it has low Michael addition reactivity, and (2) it is hydrolysed readily with a systemic half-life of less than five minutes (Jones 2002). Nevertheless, despite being classified as a weak sensitiser, numerous publications have documented cases of allergic contact dermatitis caused by MMA, as confirmed by patch testing, often along with cases caused by other (meth)acrylates (Spencer et al. 2016; Gardeen and Hylwa 2020). Several cases of allergic contact dermatitis in workers handling MMA have been recorded (see e.g. Wrangsjö et al. 2001). These cases occurred across all industry sectors that manufacture and handle MMA especially if control measures, such as use of skin protection equipment, had not been implemented (Kimber and Pemberton 2014).

#### Regulatory classification of MMA

MMA is classified as a skin irritant and sensitiser and respiratory irritant in the current EU Harmonised Classification and Labelling (CLH) system (https://www.echa.europa.eu/web/guest/information-on-chemicals/cl-inventory-database/-/discli/details/104369). While currently not designated as a respiratory sensitiser, in March 2021, the ECHA Committee for Risk Assessment (RAC) proposed to add Respiratory Sensitisation Category 1 to the CLH of MMA (ECHA RAC 2021). In the ECHA RAC (2021) Opinion, it is stated that since there are no validated experimental animal assays with which to assess respiratory sensitisation, the data considered for the evaluation rely on case reports, worker health studies and cases reported to national health surveillance databases.

Specifically, Suojalehto et al. (2020) reported the key study cited to support the proposal in the ECHA RAC (2021) Opinion to classify MMA as a respiratory sensitiser. Suojalehto et al. presented the findings from a retrospective observational study that included subjects from 20 tertiary centres who had been diagnosed with OA, all of whom had been confirmed by SIC testing, yielding positive outcomes for acrylates (*n* = 55) or other low molecular weight agents (*n* = 418) including isocyanates (*n* = 125). There was limited additional information on chemical-specific exposure. Subsequently, Suojalehto and colleagues transmitted additional information to ECHA on six subjects that “*had been predominantly exposed to MMA and tested positive specifically for this substance… ‘predominantly’ meaning that the main component of the products used was MMA, as opposed to mixed-exposures with other (meth)acrylates*” (page 8–9 of ECHA RAC 2021). Two of these six subjects were dentists, three were dental and medical prosthesis technicians, and one was a nail beautician that was reported to use MMA for acrylic nails (page 9 of ECHA RAC 2021). The ECHA RAC (2021) Opinion states that experts were able to verify from the original product information that three of the subjects had been exposed to “*two-component, self-curing methacrylate products containing MMA as their main ingredient*” and the other three to “*two-component MMA products to make prostheses*” (page 9 of ECHA RAC 2021). The specific composition of the applied products was not disclosed; i.e. beyond these citations, the ECHA RAC (2021) Opinion did not include any further details on the applied products. From amongst the six subjects, three patients had late asthmatic reactions, two patients, a dual (biphasic) positive response and one, an early reaction (page 9 of ECHA RAC 2021). No indication was provided of the estimated levels of exposure to MMA or likely relative exposure compared with other workplace environments where MMA exposure would be expected.

The Panel noted that the ECHA RAC (2021) Opinion does not specify considerations for the selection of the Suojalehto et al. (2020) study in particular, nor for the six specific case reports from the broader database, or for their evaluation using a WoE evaluation as specified in the EU CLP Regulation (EP and Council 2008) and subsequent guidance (ECHA 2017b).

### Panel discussions on MMA as an example for evidential requirements for the regulatory hazard and risk assessment of respiratory sensitisers

The database available for sensitising and respiratory effects of MMA encompasses a broad spectrum of different types of studies and information sources (ECHA RAC 2021). Though not apparent in the ECHA RAC Opinion, as discussed in further detail in  “Application of formal WoE frameworks for the assessment of respiratory sensitisers”, use of predefined, contextually relevant considerations would contribute to increasing transparency, consistency and objectivity in selecting studies and lines of evidence, in establishing the relevance and reliability of the data within a line of evidence (e.g. human, animal, in vitro) and in weighting and integrating the data from all lines of evidence. The most important evidence available for MMA has been derived from case reports, worker health studies and cases reported to national health surveillance databases. Therefore, application of predefined criteria to evaluate the sufficiency of human data implemented in Chapter R.7.3.10.2 of the ECHA (2017a) guidance ( “Prevalence/incidence of occupational asthma in epidemiological studies”) for MMA would increase transparency on e.g. substance identity, the extent of exposure and the presence of confounding factors. While available case reports and worker health studies evaluating respiratory effects of MMA may not fulfil all specified criteria, limitations are normally addressed during WoE evaluations through weighting of those studies in which confidence is greatest.

In evaluating the evidence from different (types of) human studies, the prevalence and incidence from different populations and sectors should be compared and weighted while also considering whether study methodology and estimation of prevalence values have been adequately reported (“ Application of formal WoE frameworks for the assessment of respiratory sensitisers”). Based upon the human database considered in the ECHA (2021) RAC Opinion, in those sectors in which there are anticipated high exposures to MMA for long periods of time (e.g. cast acrylic sheets industry and floor coating sector;  “Background on use and hazard profile”), there have been no reports of OA caused by MMA. The six cases of focus in the ECHA (2021) RAC Opinion include two dentists, three dental and medical prosthesis technicians, and a nail beautician (“ Regulatory classification of MMA”). Information from the published literature indicates that dental technicians and nail beauticians may be exposed to a wide range of dusts and (volatile) chemicals and thus to much lower levels of airborne MMA than workers in the cast acrylic sheets industry or floor coating sector. For example, dental restorative materials (referred to as two-component “acrylic” or “MMA-based” cements) may contain many other (meth)acrylates in addition to or instead of MMA (Henriks-Eckerman et al. 2004). Similarly, artificial nails contain a broad spectrum of (meth)acrylates (Kanerva et al. 1996; Lazarov 2007). Furthermore, in dental settings, dental technicians, but not normally dentists and dental nurses, are generally the only professionals exposed to MMA (Aalto-Korte et al. 2007). These mixed exposures could explain why OA was seen in the dental sector, but not in the other sectors. During a structured, formal WoE evaluation, the contribution of negative epidemiological studies in large cohorts yielding no evidence of OA would be appropriately weighted based on their quality (relevance and reliability), including statistical power.

To the extent permitted by the available data, the formal WoE evaluation would also consider the physical and chemical properties of MMA and related compounds (e.g. acrylates), their potential to cause (skin) sensitisation as well as their toxicokinetic properties. This might also help clarify the potential association between potency for dermal sensitivity and potential to cause respiratory sensitisation. Consideration of the extent of the evidence on the development of OA upon exposure to known respiratory sensitisers would additionally inform the nature of datasets considered as sufficient to be classified as respiratory sensitisers.

The Panel also discussed possible mechanisms of respiratory effects caused by MMA while noting that this information is not necessary for classification as respiratory sensitiser. These discussions also considered that asthma may generally also be caused by neurosensory events (“ Pathophysiology of occupational asthma and of allergic reactions”). MMA is classified as a respiratory irritant within the EU (“Regulatory classification of MMA”), and some Panel Members suggested that the irritant MoA may be the most important mechanism for the development of respiratory effects caused by MMA, but that this effect could differ among individuals. A preliminary possible ranking of the likelihood for the MoA of MMA associated respiratory effects was proposed: irritation was suggested to rank highest, followed by the neurosensory reaction (due to the strong odour of MMA), followed by respiratory sensitisation. Justification for this ranking would be the overall low incidence of OA in populations that may be exposed to MMA (see above) and the evidence that MMA is a weak dermal sensitiser. It was questioned, however, whether the evidence was sufficient to support this (or any other) ranking. In particular, the possibility of immune-mediated respiratory responses could not be excluded based upon the current database. The Panel agreed that further work would be needed to test this hypothetical ranking.

## Recommendations from the Expert Panel: regulatory hazard and risk assessment of respiratory sensitisers

The Panel recommended that classifications for respiratory sensitisation should be based on formal (systematic and robust) WoE evaluations of the available evidence on both respiratory effects and sensitisation potential. This would increase transparency and facilitate objectivity in the different steps of data collection, selection and evaluation of the individual studies and form a basis for the integration and weighting of a broad range of different lines of evidence. Additionally, formal WoE evaluations would facilitate more consistent consideration of the confidence in the level of certainty of the causality of associations between exposure to specific chemicals and respiratory sensitisation.

As a starting point to draw up a formal WoE evaluation framework for the assessment of respiratory sensitisation, the Panel recommended to consider the structure of the ECHA WoE / uncertainty templates ( “Background to WoE evaluations”), as well as predefined, contextually relevant considerations for the evaluation of individual studies and/or contributing data. Integration of different lines of evidence is addressed through predefined considerations based on (an adaptation of) the modified Bradford Hill considerations described in the WHO IPCS Framework on MoA and Species Concordance Analysis, taking into account the collective evidence base to address, for example, biological plausibility, empirical support (dose–response and temporal concordance) and analogy (Meek et al. 2014a, b).

Predefined considerations for the evaluation of the quality of individual studies such as case reports relevant to the assessment of causality for respiratory sensitisation would be helpful. These predefined considerations should address amongst other items, the adequacy of the reporting of the specific substances or mixtures to which the workers were exposed, the frequency and levels of exposure as well as potentially confounding factors (Prevalence/incidence of occupational asthma in epidemiological studies”). The Panel suggested that it might be helpful to involve experts in the field of respiratory sensitisation in establishing common ontology and the definition of criteria to establish the quality of data from case reports and clinical studies (e.g. by analogy to the Klimisch et al. (1997) criteria for the evaluation of toxicity data). When predefining contextually relevant considerations for integration across the database, it could be considered whether or not there is sufficient information to rank their relative contribution to the overall WoE evaluation for causality (Meek et al. 2014b).

Further predefined considerations for systematic and transparent evidence identification and assimilation might also address the importance of other potentially relevant data sources including in silico modelling, in chemico and in vitro assays as well as animal studies. The Panel also suggested that a formal, systematic WoE evaluation of the available data on respiratory effects and sensitisation caused by a range of chemicals (such as MMA) would be helpful in delineating general selection and decision considerations for the classification of respiratory sensitisers.

Integration of available evidence in a formal WoE evaluation facilitates transparent analysis of the uncertainties and identification of critical data gaps to permit or refine assessment. Depending on the outcome, relevant additional data might be generated in prospective animal studies, e.g. using the approach described in Pollaris et al. (2019), and/or in human immunological studies. However, the Panel also noted that formal WoE evaluation of the available evidence cannot resolve fundamental uncertainties such as the pathophysiology of respiratory sensitisation.

## Data Availability

This is a review article that does not contain original data. All cited data have been appropriately referenced.

## Appendix: Glossary

Adverse outcome pathway (AOP): A linear sequence of events commencing with initial interaction(s) of a stressor with a biomolecule within an organism that causes a perturbation in its biology (i.e. molecular initiating event), which can progress through a dependent series of intermediate key events and culminate in an adverse outcome considered relevant to risk assessment or regulatory decision-making (Ankley et al. 2010; OECD 2017a, b).

Asthma: “*A long-term condition affecting children and adults. The air passages in the lungs become narrow due to inflammation and tightening of the muscles around the small airways. This causes asthma symptoms: cough, wheeze, shortness of breath and chest tightness. These symptoms are intermittent and are often worse at night or during exercise. Other common ‘triggers’ can make asthma symptoms worse. Triggers vary from person to person, but can include viral infections (colds), dust, smoke, fumes, changes in the weather, grass and tree pollen, animal fur and feathers, strong soaps, and perfume*”; https://www.who.int/news-room/fact-sheets/detail/asthma.

Case report: A detailed report of the diagnosis, treatment, and follow-up of an individual patient. Case reports also contain some demographic information about the patient (for example, age, gender, ethnic origin); https://www.cancer.gov/publications/dictionaries/cancer-terms/def/case-report. A case report is a detailed report of the symptoms, signs, diagnosis, treatment, and follow-up of an individual patient. Case reports usually describe an unusual or novel occurrence and as such, remain one of the cornerstones of medical progress and provide many new ideas in medicine (Heart Views 2017).

Clinical study: A study designed to measure the safety, efficacy, and appropriate dosage of a new drug or biological (https://medical-dictionary.thefreedictionary.com/clinical+study). Clinical study involves research using human volunteers (also called participants) that is intended to add to medical knowledge (https://clinicaltrials.gov/ct2/about-studies/learn).

Dual asthmatic reaction (SIC test): Dual/biphasic reactions: a combination of immediate and late reactions (Vandenplas et al. 2014; see also early asthmatic reaction and late asthmatic reaction).

Early asthmatic reaction (SIC test); also: immediate asthmatic reaction: Onset of the asthmatic reaction during exposure or a few minutes after the end of exposure and recovery over 1–2 h (Vandenplas et al. 2014).

EC3 value (Local Lymph Node Assay): The amount of test chemical required to elicit a threefold increase in proliferative activity compared with concurrent vehicle control values (Basketter et al. 1999). Following ECETOC (2008), skin sensitisers are established as weak if they yield EC3 values ≥ 10%.

Epidemiological study: A study that compares two or more groups of people who are alike except for one factor, such as exposure to a chemical or the presence of a health effect; the investigators try to determine if any factor is associated with the health effect (adapted from https://medical-dictionary.thefreedictionary.com/epidemiologic+study).

Incidence: “*The rate of new cases or events over a specified period for the population at risk for the event. In medicine, the incidence is commonly the newly identified cases of a disease or condition per population at risk over a specified timeframe*” (https://www.ncbi.nlm.nih.gov/books/NBK430746/).

Late asthmatic reaction (SIC test): Onset of the asthmatic reaction later than 2 h after the exposure (Vandenplas et al. 2014).

Line of evidence (in WoE evaluation): Depending on the formulated hypothesis, a line of evidence conjoins different (types of) studies that are used to critically test the hypothesis (Bridges et al. 2017).

Mode-of-action (MoA): *“A biologically plausible sequence of key events leading to an observed effect supported by robust experimental observations and mechanistic data. A MoA describes key cytological and biochemical events – that is, those that are both measurable and necessary to the observed effect – in a logical framework”* (WHO 2009; Definitions page A-25).

Physiologically based kinetic modelling: modelling to describe the fate of a substance in the organism by mathematical equations. Physiologically based kinetic modelling is a more general term for specific ones used so far, such as physiologically based pharmacokinetic, physiologically based biokinetic, or physiologically based toxicokinetic modelling (adapted from Laroche et al. 2018).

Prevalence: “*The proportion of a population who have a specific characteristic in a given time period*” (https://www.nimh.nih.gov/health/statistics/what-is-prevalence).

Read-across: A technique for predicting endpoint information for the target substance using available data for the same endpoint from the source substance(s) (adapted from ECHA 2017c).

Respiratory sensitisation: An immunological state of the respiratory tract that results from specific adaptive immune responses to antigenic exposure, leading to heightened immunological responsiveness after subsequent exposures to the sensitising antigen. In turn, such heightened respiratory tract responsiveness can result in allergic reactions characterised by airway obstruction, nonspecific bronchial hyperreactivity and inflammation that may present clinically as allergic rhinitis, asthma, and extrinsic allergic alveolitis (Borak et al. 2011; citing Kimber et al. 2007; Boverhof et al. 2008; Isola et al. 2008). “*Hypersensitivity of the airways occurring after inhalation of a substance or mixture*” (United Nations 2021).

Respiratory sensitiser: “*An agent that will lead to hypersensitivity of the airways following inhalation exposure to that agent. Respiratory sensitisation (or hypersensitivity) is a term that is used to describe asthma and other related respiratory conditions (rhinitis, extrinsic allergic alveolitis), irrespective of the mechanism (immunological or non-immunological) by which they are caused. In contrast, skin allergy is based on an immunological mechanism*” (ECHA 2017a).

Sensitisation: “…*includes two phases: the first phase is induction of specialised immunological memory in an individual by exposure to an allergen. The second phase is elicitation, i.e. production of a cell-mediated or antibody-mediated allergic response by exposure of a sensitised individual to an allergen*” (United Nations 2021).

Skin sensitisation: “*an allergenic response occurring after skin contact with a substance or mixture*” (United Nations 2021).

Specific inhalation challenge test (SIC test): The controlled exposure of a patient, under laboratory conditions, to an agent encountered in their workplace (Vandenplas et al. 2014).

Weight-of-evidence (WoE): “*The extent to which evidence supports possible answers to a scientific question*”; accordingly, the WoE evaluation is “*a process in which evidence is integrated to determine the relative support for possible answers to a scientific question*” (EFSA SC 2017).
